# Risks of Maternal Obesity in Pregnancy: A Case-control Study in a Portuguese Obstetrical Population

**DOI:** 10.1055/s-0039-3400455

**Published:** 2019-12

**Authors:** Patrícia Alves, Maria Filipa Malheiro, João Cavaco Gomes, Tiago Ferraz, Nuno Montenegro

**Affiliations:** 1 Department of Obstetrics and Gynecology, Centro Hospitalar São João, Porto, Portugal; 2 Department of Gynecology and Obstetrics, Centro Hospitalar de Trás-os-Montes and Alto Douro, Vila Real, Porto, Portugal; 3 Medicine Faculty, Universidade do Porto, Porto, Portugal; 4 I3S Innovation in Health and Investigation Institute, Universidade do Porto, Porto, Portugal; 5 Epidemology Research Unit, Institute of Public Health, Universidade do Porto, Porto, Portugal

**Keywords:** cesarean section, diabetes gestational, fetal macrosomia, obesity, high-risk pregnancy, cesariana, diabetes gestacional, macrossomia fetal, obesidade, gravidez de alto risco

## Abstract

**Objective** The present study aims to understand to what extent obesity is related to adverse maternal, obstetrical, and neonatal outcomes in a Portuguese obstetrical population.

**Methods** A retrospective case-control study was conducted at the Department of Obstetrics of a differentiated perinatal care facility. The study compared 1,183 obese pregnant women with 5,399 normal or underweight pregnant women for the occurrence of gestational diabetes, hypertensive pregnancy disorders, and preterm birth. Mode of delivery, birthweight, and neonatal intensive care unit (ICU) admissions were also evaluated. Mean blood glucose values were evaluated and compared between groups, in the first and second trimesters of pregnancy. Only singleton pregnancies were considered.

**Results** The prevalence of obesity was 13.6%. Obese pregnant women were significantly more likely to have cesarean sections (adjusted odds ratio [aOR] 2.0, *p* < 0.001), gestational diabetes (aOR 2.14, *p* < 0.001), hypertensive pregnancy disorders (aOR 3.43, *p* < 0.001), and large-for-gestational age or macrosomic infants (aOR 2.13, *p* < 0.001), and less likely to have small-for-gestational age newborns (aOR 0.51, *p* < 0.009). No significant differences were found in terms of preterm births, fetal/neonatal deaths, low birthweight newborns, and neonatal ICU admissions among cases and controls. Maternal obesity was significantly associated with higher mean blood glucose levels, in the first and second trimesters of pregnancy.

**Conclusion** Obesity is associated with increased risks of adverse pregnancy and neonatal outcomes. These risks seem to increase progressively with increasing body mass index (BMI) class. Female obesity should be considered a major public health issue and has consequences on maternal-fetal health.

## Resumo

**Objetivo** O presente estudo pretende avaliar em que medida a obesidade influencia os desfechos maternos, obstétricos e neonatais em uma população obstétrica portuguesa.

**Métodos** Um estudo caso-controle retrospectivo foi realizado no departamento de obstetrícia de um centro perinatal diferenciado. O estudo comparou 1.183 grávidas obesas com 5.399 grávidas normoponderais ou com baixo peso para a ocorrência de diabetes gestacional, doenças hipertensivas da gravidez e parto pré-termo. Via de parto, peso ao nascimento e admissão na unidade de cuidados neonatais também foram avaliados. Os valores glicêmicos médios foram avaliados e comparados entre os dois grupos, no primeiro e segundo trimestres de gravidez. Apenas as gravidezes unifetais foram avaliadas.

**Resultados** A prevalência da obesidade foi de 13.6%. As grávidas obesas tiveram risco significativamente superior a ter uma cesariana (odds ratio ajustado [Ora] 2.0, *p* < 0.001), diabetes gestacional (ORa 2.14, *p* < 0.001), doenças hipertensivas da gravidez (ORa 3.43, *p* < 0.001), recém-nascidos grandes para a idade gestacional ou macrossômicos (ORa 2.13, *p* < 0.001) e menor probabilidade de ter recém-nascidos pequenos para a idade gestacional (ORa 0.51, *p* < 0.009). Não houve diferença estatisticamente significativa quanto aos partos pré-termo, mortes fetais/neonatais, baixo peso ao nascer e admissão à unidade de cuidados intensivos neonatais. O odds ratio foi ajustado para a idade, número de gestações, paridade, ganho ponderal, doenças hipertensivas da gravidez e diabetes gestacional. A obesidade materna esteve significativamente associada a valores glicêmicos médios superiores, no primeiro e segundo trimestres de gravidez.

**Conclusão** A obesidade está associada a maior risco de desfechos adversos na gravidez e neonatais. Este risco parece aumentar progressivamente com o aumento do índice de massa corporal (IMC). A obesidade feminina deve ser considerada um importante problema de saúde pública e que tem repercussões na saúde materno-fetal.

## Introduction

The World Health Organization (WHO) considers obesity a worldwide epidemic and one of the greatest public health challenges of the 21^st^ century. According to the WHO, in 2016, across Europe, 24.5% of women aged ≥ 18 years old were obese.^1^ In the same year, in Portugal, the prevalence was 21.2% and represented a 3-fold rise since 1975 (6.8%).^2^ ^3^

The etiology of obesity is multifactorial and complex. Obesity is related to genetic predisposition, physiological changes to the endocrine system of the body, potential genetic contributions over generations, cultural beliefs, and socioeconomic issues.^4^

Obesity has a major impact on both morbidity and mortality. Obesity is a risk factor for type 2 diabetes mellitus (DM), hypertension, dyslipidemia, and coronary heart disease. Also, obesity decreases quality of life because of associated mood disorders, such as anxiety and depression, and aggravated osteoarticular complaints.^4^ ^5^

In pregnancy, obesity is a risk factor for adverse maternal, obstetrical, and fetal/neonatal outcomes, contributing to prolonged hospitalization periods, both for the mother and the baby.^4^ ^6^ Obesity increases risks of venous thromboembolism, gestational diabetes, preeclampsia, dysfunctional labor, cesarean delivery, postpartum hemorrhage, wound infection, miscarriage, fetal/neonatal death, and abnormal fetal growth, either macrosomia or growth restriction.^7^ ^8^ Moreover, children of obese parents have a two to three times higher risk of becoming obese adults. It seems that the *in utero* environment plays a causative role in this vicious cycle.^4^

Obesity in pregnancy is defined as a body mass index (BMI) equal to or greater than 30 Kg/m^2^ at the first prenatal visit. It is further subclassified in: class I (30.0–34.9 Kg/m^2^), class II (35.0–39.9 Kg/m^2^), and class III (≥ 40 Kg/m^2^).^8^

The aim of the present study was to understand to what extent obesity is related to adverse maternal, obstetrical, and neonatal outcomes in a Portuguese obstetrical population.

## Methods

### Study Design

This retrospective case-control study was conducted using 4 years of data of women who gave birth at the Department of Obstetrics of a differentiated perinatal care University Hospital, between January 2013 and December 2016. Only singleton pregnancies were considered. Ethics approval was obtained from the Ethics Committee of our hospital.

A total of 9,371 participants were selected. Information about BMI at the first prenatal visit was lacking from 659 medical records and these pregnant women were promptly excluded. The remaining 8,712 pregnant women were categorized according to WHO BMI categories, based on the registered weight at the first prenatal visit.^8^ Overweight women (*n* = 2,130) were further excluded to get a more accurate comparison, because overweight pregnant women are predisposed to obesity. The final analysis included 6,582 singleton pregnancies: A group of 1,183 obese pregnant women (cases) were compared with a group of 5,399 normal or underweight pregnant women (controls) for maternal, obstetrical, and neonatal outcomes.

### Data Collection

Maternal, obstetrical, and perinatal data from singleton pregnant women who gave birth in the maternity facility, irrespective of type of pregnancy follow-up, were collected from Obscare (Virtual Care, System for life, Porto, Portugal), an institutional medical record software for obstetricians and pediatricians.

### Variables Description

Information was collected on women's age, parity, weight (at the first and last prenatal visits), and BMI (Kg/m^2^) at the first prenatal visit. The weight gain was calculated from the difference in weight between the last and first prenatal visits and used as a continuous variable.

Gestational diabetes was diagnosed according to the International Association of the Diabetes and Pregnancy Study Groups criteria (IADPSGC).^9^ Hypertensive pregnancy disorders (gestational hypertension and preeclampsia) were considered when maternal blood pressure was ≥ 140 mm Hg (systolic) or ≥ 90 mm Hg (diastolic) on two occasions, at least 4 hours apart, after 20 weeks of gestation, in a woman with a previously normal blood pressure.^10^ Other variables studied were delivery mode, fetal demise, gestational age at birth, birth weight, Apgar score, neonatal intensive care unit admission, and neonatal death. Preterm birth was classified as extreme preterm (24–28 weeks), very preterm (29–32 weeks), and moderate/late preterm (32–36 weeks). After this categorization of preterm birth, it has also been grouped to be estimated as a dichotomous variable – preterm and term births. An updated and validated Portuguese birthweight chart was used to obtain birthweight percentiles.^11^ Newborns were classified as small for gestational age (SGA) when birth weight was < 10^th^ percentile for the gestational age, and as large for gestational age (LGA) when the birth weight was ≥ 90^th^ percentile. Low birthweight was considered when infants weighed ≤ 2,500 g, and macrosomia when they weighed ≥ 4,000 g. Gestational diabetes, hypertensive pregnancy disorders, delivery mode, fetal demise, neonatal intensive care unit admission, and neonatal death were evaluated as dichotomous variables.

Blood glucose values, in the first (fasting) and second (fasting, 1 and 2 hours after 75 g glucose load) trimesters of pregnancy were evaluated and compared between groups, as continuous variables.

### Statistical Analysis

Descriptive statistics were performed for demographic, clinical, and laboratory data. Mean and standard deviation (SD) were calculated for normally distributed variables. For group comparisons, parametric (t test student and analysis of variance [ANOVA]), and nonparametric tests (Mann-Whitney test) were used, as appropriate, for continuous variables, and the Pearson Chi^2^ test for categorical variables.

Logistic or linear regression analysis, as appropriate, for univariate and multivariate models were used for each of the outcomes. Odds ratio (OR) was adjusted for age, number of gestations, parity, weight gain, hypertensive pregnancy disorders, and gestational diabetes. All of the results were considered significant if the *p-value* was < 0.05. Statistical analyses were performed using Stata version 12.1 (Stata Corp, College Station, TX, USA).

## Results

The prevalence of obesity in the obstetrical population studied, as registered in the first prenatal visit, was 13.6%, and the mean BMI was 24.7 Kg/m^2^ (Table 1).

**Table 1:** Distribution of pregnant women by body mass index category

BMI category (Kg/m^2^)	Number (prevalence %)
Underweight (< 18.5)	325 (3.7)
Normal weight (18.5–24.9)	5074 (58.2)
Overweight (25.0–29.9)	2130 (24.5)
Obesity	1183 (13.6)
Obesity class I (30.0–34.9)	819 (9.4)
Obesity class II (35.0–39.9)	268 (3.1)
Obesity class III (≥40)	96 (1.1)

Abbreviation: BMI, body mass index.

Table 2 summarizes maternal characteristics. The obese group of women was significantly older, more frequently multiparous, and gained less weight during pregnancy than normal or underweight women.

**Table 2:** Maternal characteristics

	Obesity group (*n* = 1,183)	Control group (*n* = 5,399)	OR	*p-value*
Age (years old) (mean, SD)	31.5 (5.5)	30.7 (5.6)	–	< 0.001
Age > 35 years old (%)	32	26	1.37 (1.2–1.57)	< 0.001
Number of gestations (n^‡^)	2.1	1.8	–	< 0.001
Nulliparous (%)	35	47.5	0.59	< 0.001
BMI (kg/m^2^) (mean, SD)	34.1 (3.9)	21.7 (4.9)	–	< 0.001
Weight gain (kg) (mean, SD)	10.5 (6.8)	14.3 (4.9)	–	< 0.001

Abbreviations: BMI: body mass index; kg: kilograms; n: number; OR: odds ratio; SD: standard deviation.

Obese women had a significantly higher prevalence of gestational diabetes (17.6% versus 5.5%, adjusted odds ratio [aOR] 2.14; 95% confidence interval [CI]: 1.53–3.00) and hypertensive pregnancy disorders (9.0% versus. 2.6%, aOR 3.43; 95%CI: 2.33–5.12). Concerning the mode of delivery, the cesarean section rate was significantly more frequent in the obesity group compared with the control group (35.3% versus 24.4%). After adjusting for confounders, obese pregnant women had twice the odds of delivering by cesarean (aOR 2.0; 95%CI: 1.64–2.47) compared with normal or underweight women. The difference was even more significant among primigravidae (aOR 2.27; 95%CI: 1.65–3.11). No differences were found in preterm birth rates between the 2 groups (8.3% versus 7.1%, obesity and control groups respectively, *p* = 0.17). The mean birth weight was significantly higher in the obese group (3,226 ± 531 g) compared with the control group (3,132 ± 506 g). Large for gestational age and macrosomic newborns were significantly more prevalent among obese women (12.8% versus 6.9%, aOR 2.13; 95%CI: 1.54–2.96; and 6.4% versus 3.2%, aOR 2.94, 95%CI: 1.95–4.45, respectively), even when adjusted for age, parity, weight gain, gestational diabetes, and hypertension. Considering the morbidly obese pregnant women (BMI ≥ 40 Kg/m^2^), the risk of having a macrosomic newborn was > 9 times higher than that of a normal or underweight pregnant woman (aOR 9.5; 95%CI: 3.7–24.6) (Table 3). In contrast, obese pregnant women had significantly fewer SGA newborns (9.7% versus. 12.1%, *p* = 0.009), but no statistical significant difference was observed for the low birthweight variable.

**Table 3:** Risks of maternal, obstetrical and neonatal outcomes according to obesity class

	Control	Obesity class I	Obesity class II	Obesity class III
	aOR	aOR (95%CI)	aOR (95%CI)	aOR (95%CI)
Gestational diabetes	Ref	1.98 (1.35–2.9)	2.42 (1.37–4.26)	2.1 (0.92–4.80)
Hypertensive pregnancy disorders	Ref	3.52 (2.27–5.45)	2.54 (1.10–5.85)	6.38 (2.49–16.35)
Cesarean section	Ref	1.78 (1.41–2.25)	2.61 (1.77–3.85)	3.19 (1.79–5.71)
SGA	Ref	0.59 (0.41–0.84)	0.62 (0.35–1.10)	0.1 (0.02–0.50)
Low-birthweight infant	Ref	0.71 (0.49–1.03)	0.45 (0.24–0.85)	0.08 (0.20–0.38)
LGA	Ref	1.69 (1.17–2.44)	3.93 (2.36–6.60)	7.0 (3.42–14.30)
Macrosomia	Ref	2.25 (1.35–3.74)	5.02 (2.47–10.20)	9.53 (3.70–24.60)

Abbreviations: aOR, adjusted odds ratio; LGA, Large for gestational age; Ref, reference value; SGA, Small for gestational age.

All variables are adjusted for age, number of gestations, parity, weight gain, hypertensive pregnancy disorders, gestational diabetes.

According to a local institutional policy, immediate postpartum umbilical cord blood gas analysis is performed only in cases of suspected fetal hypoxia/acidosis. In a total of 4,388 tests, fetal acidemia (pH < 7.2 in umbilical artery) was more frequently found in the obese group of women (8.9% versus 6.7%, *p* = 0.008), but no differences were found among the groups for severe acidemia (pH < 7.05 in the umbilical artery). Apgar score < 7 at 5 minutes was identical in both groups (1.2%, *p* = 0.95), and even though more newborns from obese women were admitted to the neonatal intensive care unit (ICU), the difference was not statistically significant (6.8 versus 5.6%, *p* = 0.23).

Fetal and neonatal death rates were not significantly different between obese pregnant women (*n* = 4, 0.3%) compared with the normal or underweight pregnant woman (*n* = 32, 0.6%).

Table 3 presents maternal, obstetrical, and neonatal outcomes according to obesity class. The risk of gestational diabetes, hypertensive pregnancy disorders, cesarean delivery, LGA, and macrosomic infants increased with increasing BMI class. In contrast, the odds of low birth weight and SGA infants decreased with increasing BMI class.

Blood glucose levels were significantly higher for obese pregnant women compared with normal or underweight women (*p* < 0.001) (Table 4). Also, mean blood glucose levels were found to progressively increase with increasing class of obesity (Table 5).

**Table 4:** Blood glucose levels (mg/dL)

	Obesity group (mean, SD^§^)	Control group (mean, SD)	*p-value*
1^st^ trimester fasting	83.5 (9.0)	79.3 (7.2)	< 0.001
2^nd^ trimester fasting	77.8 (9.4)	73.8 (17.4)	< 0.001
2^nd^ trimester 1-hour after OGTT¶	127.6 (31.3)	113.9 (29.3)	< 0.001
2^nd^ trimester 2 hours after OGTT¶	109 (26.6)	98.6 (24.9)	< 0.001

Abbreviation: SD, standard deviation.

¶ OGTT (oral tolerance glucose test), 75 gr glucose load (fasting, 1 and 2 hours after), at 24–28 weeks.

**Table 5:** Blood glucose levels (mg/dL) according to obesity class

	Control (χ ± SD‡)	Obesity class I (χ ± SD)	Obesity class II (χ ± SD‡)	Obesity class III (χ ± SD)	*p-value* (ANOVA)
1^st^ trimester fasting	79.3 (7.2)	82.5 (8.6)	85.5 (10.1)	86.1 (8.5)	< 0.001
2^nd^ trimester fasting	73.8 (17.4)	77.5 (9.8)	77.9 (8.1)	80.0 (7.5)	< 0.001
2^nd^ trimester 1-hour after OGTT†	113.9 (29.3)	127 (32.2)	128.1 (28.5)	132.2 (28.7)	< 0.001
2^nd^ trimester 2 hours after OGTT†	98.6 (24.9)	108.8 (29.9)	109.8 (27.3)	109.1 (22.1)	< 0.001

Abbreviations: ANOVA, .Analysis of variances model; SD, standard deviation.

† OGTT (oral tolerance glucose test), 75 gr glucose load (fasting, 1 and 2 hours after), at 24–28 weeks.

‡ χ: mean.

## Discussion

Our study reported a 13.6% prevalence of maternal obesity in a Portuguese population of 6,582 singleton pregnancies. So far, this information concerning specifically a Portuguese obstetrical population was unavailable.

Obese pregnant women included in the analysis were significantly older and more frequently multiparous than normal or underweight women, reflecting a progressive tendency for weight gain with increasing age and parity.^12^

Excessive weight gain during pregnancy is a hallmark of poor metabolic control and favors adverse pregnancy outcomes.^7^ ^8^ The 2013 American College of Obstetricians and Gynecologists (ACOG)^13^ recommendations for weight gain during pregnancy for obese women was between 5 and 9.1 Kg. In our study, the average weight gain during pregnancy in obese women exceeded the maximum allowed (10.5 Kg). This finding should alert Portuguese physicians involved in women's and antenatal medical care to specifically address prevention and management of obesity, through nutritional changes, physical conditioning, and promotion of healthy lifestyle changes.

The results from the present study support the fact that maternal obesity is a major risk factor for adverse pregnancy and perinatal outcomes.

We demonstrated that gestational diabetes is twice as likely for obese pregnant women, which is somewhat lower than the odds reported in the literature (OR 3.6–7.5).^7^ ^14^ We also demonstrated increases in mean blood glucose levels, during the first and second trimesters of pregnancy, with increasing BMI class. The HAPO study explained this metabolic change through increases in insulin resistance with higher BMI values and suggested that gestational diabetes and obesity seem to share common metabolic features, such as increased insulin resistance, hyperglycemia, and hyperinsulinemia.^15^

Hypertensive pregnancy complications were also more likely to occur in obese pregnant women and, specifically, in the morbidly obese pregnant women (BMI > 40 Kg/m^2^) (aOR 6.38; 95%CI: 2.49–16.35) (Table 3). Analogously, an Australian study demonstrated that obese pregnant women had 3 times the odds of having a hypertensive disorder during pregnancy, and the risk was even higher among the morbidly obese (OR 4.87; 95% CI: 3.27–7.24).^12^

In our study, maternal obesity was an independent risk factor for delivering macrosomic and LGA infants. In contrast, maternal obesity reduced the risk of SGA or low birthweight newborns.^16^

Cesarean delivery rates have been increasing over the past 30 years in both developed and developing countries. In Portugal, by the year 2011, cesarean sections accounted for 35% of total deliveries.^17^ These high rates led to the creation, in 2013, of a National Committee for Safe Motherhood and Newborn Health to try to counteract this tendency toward an unnatural way of birth.^18^ In accordance with the published literature, our study demonstrated a negative influence of maternal obesity on delivery mode, favoring cesarean section (Table 3).^7^ ^19^ ^20^ Obese pregnant women were two times more likely to have cesarean sections compared with normal or underweight women, and the odds were three times higher for the morbidly obese. This difference persisted even when considering only primigravidae, which excluded the effect caused by obstetric history, such as cesarean section. So, the increase in maternal obesity further contributes to the present difficulty in achieving the 2015 WHO's goal for a cesarean section rate of 10 to 15%.^17^

In the literature, there is controversy regarding the association between maternal obesity and preterm birth.^7^ ^12^ ^16^ Our study found similar rates of preterm birth for both obese and normal or underweight women.

Our study did not demonstrate increased rates of fetal or neonatal death in the obese group of women, which is different from what is already published.^7^ ^8^ ^16^ This result may be explained by the number of obese women, which may have been insufficient to evaluate infrequent adverse obstetrical and neonatal events such as fetal or neonatal death.

The present study has further limitations. First, the present findings were derived from a single maternity hospital in Portugal, so that, despite the large sample, limited generalization is possible. Also, the study is a retrospective comparative analysis of maternal, obstetrical, and neonatal data, and some data could not be collected. For example, in an unknown percentage of medical records, maternal weight, as registered in the first prenatal visit, may not have been objectively measured, leading to self-reported errors concerning this important variable.^16^

This is the first Portuguese study that specifically addressed maternal, obstetrical, and neonatal outcomes in a population of singleton obese pregnant women and compared them with those of normal or underweight pregnant women.

## Conclusion

In accordance with the published literature, the present retrospective case-control study was able to demonstrate that obesity is associated with increased odds of adverse pregnancy and neonatal outcomes, such as gestational diabetes, hypertensive pregnancy disorders, cesarean section, macrosomia, and LGA newborns. Moreover, the occurrence of adverse outcomes increased progressively with increasing BMI class. To conclude, the results of our study reinforce the fact that it is imperative to consider female obesity as a major public health issue and to take measures to prevent and treat this condition, specifically among woman of childbearing age.
